# The Impact of Microbiome and Microbiota-Derived Sodium Butyrate on *Drosophila* Transcriptome and Metabolome Revealed by Multi-Omics Analysis

**DOI:** 10.3390/metabo11050298

**Published:** 2021-05-06

**Authors:** Fan Zhou, Biaodi Liu, Xin Liu, Yan Li, Luoluo Wang, Jia Huang, Guanzheng Luo, Xiaoyun Wang

**Affiliations:** 1 Guangdong Provincial Key Laboratory of Insect Developmental Biology and Applied Technology, Institute of Insect Science and Technology, School of Life Sciences, South China Normal University, Guangzhou 510631, China; 2019022488@m.scnu.edu.cn (F.Z.); liuxin136865812@163.com (X.L.); luoluo.wang@scnu.edu.cn (L.W.); huangj@scnu.edu.cn (J.H.); 2 State Key Laboratory of Biocontrol, School of Life Sciences, Sun Yat-sen University, Guangzhou 510275, China; liubd6@mail2.sysu.edu.cn (B.L.); liyan226@mail2.sysu.edu.cn (Y.L.)

**Keywords:** *Drosophila*, microbiome, transcriptome, metabolome

## Abstract

The host microbiome plays an important role in regulating physiology through microbiota-derived metabolites during host-microbiome interactions. However, molecular mechanism underly host-microbiome interactions remains to be explored. In this study, we used *Drosophila* as the model to investigate the influence of microbiome and microbiota-derived metabolite sodium butyrate on host transcriptome and metabolome. We established both a sterile *Drosophila* model and a conventional *Drosophila* model to demonstrate the role of sodium butyrate. Using multi-omics analysis, we found that microbiome and sodium butyrate could impact host gene expression patterns in both the sterile *Drosophila* model and the conventional *Drosophila* model. The analysis of gut microbial using 16S rRNA sequencing showed sodium butyrate treatment also influenced *Drosophila* bacterial structures. In addition, *Drosophila* metabolites identified by ultra-high performance liquid chromatography-MS/MS were shown to be affected by sodium butyrate treatment with lipids as the dominant changed components. Our integrative analysis of the transcriptome, the microbiome, and the metabolome data identified candidate transcripts that are coregulated by sodium butyrate. Taken together, our results reveal the impact of the microbiome and microbiota-derived sodium butyrate on host transcriptome and metabolome, and our work provides a better understanding of host-microbiome interactions at the molecular level with multi-omics data.

## 1. Introduction

The host microbiome plays an important role in digesting complex diets, synthesizing nutrients, and maintenance of the immune system to facilitate the survival of the host [1,2]. The number of microbial populations inhabiting the intestines is large and diverse depending on the host species. Intestinal microbes can communicate with the host through metabolites, mainly including short-chain fatty acids (e.g., acetate, propionate, and sodium butyrate). Short-chain fatty acids are the most abundant metabolites produced by the fermentation of undigested dietary fiber by intestinal microorganisms and provide the main energy source for the host intestinal cells [3]. Of all the short-chain fatty acids, sodium butyrate has received special attention because it is a key regulator that mediates the metabolic control of the microbiota [4]. In fruit fly *Drosophila melanogaster*, it has been reported that adding sodium butyrate to the diet can effectively reduce the susceptibility or short-term mortality risk during aging [5]. Thus, sodium butyrate plays a non-negligible role in the dynamic relationship between diet, gut microbiome composition, and metabolic health for all animals [6,7].

*Drosophila* has been used as a model organism for the study of host-microbiome interactions in the gut. Increasing evidence has shown that the gut microbiome can affect *Drosophila*’s physiological functions such as nutrition, metabolism and immunity [8,9]. However, the molecular mechanism underlying host-microbiome interactions in the *Drosophila* model remains to be explored. In the present study, we used *D. melanogaster* as the model to investigate the influence of microbiome and microbiota-derived metabolite sodium butyrate on host transcriptome and metabolome. We examined the impact of sodium butyrate on the host in both sterile *Drosophila* (also referred to as axenic *Drosophila* in the literature) and conventional *Drosophila*. In order to further investigate the effects of sodium butyrate on *Drosophila* at the molecular level, we detected the abundance and composition of gut microbial colonies using 16S rRNA sequencing analysis and analyzed the overall structure and metabolic activities of host transcriptional networks by integrating transcriptome and metabolome data. Finally, we carried out an integrative analysis of the transcriptome, the microbiome, and the metabolome data regulated by sodium butyrate to figure out the correlations. Our results demonstrate the impact of the microbiome- and microbiota-derived sodium butyrate on host transcriptome and metabolome, and our work provides new insights into the mechanism of host-microbiome interactions at the molecular level with multi-omics data.

## 2. Results

### 2.1. Drosophila Microbiome and Metabolites Regulate Host Gene Expression under Sterile Condition

To examine the influence of the microbiome on *Drosophila* gene expression, we first established a sterile *Drosophila* model from the embryonic stage to remove the intestinal microbes, and in parallel, we bred conventional *Drosophila* with normal intestinal microbes. To understand the potential role of sodium butyrate in *Drosophila*, we further treated the sterile *Drosophila* with or without sodium butyrate. RNA samples from conventional *Drosophila*, sterile *Drosophila,* and sterile *Drosophila* treated with sodium butyrate were extracted for RNA sequencing and data analysis.

**Figure 1:**
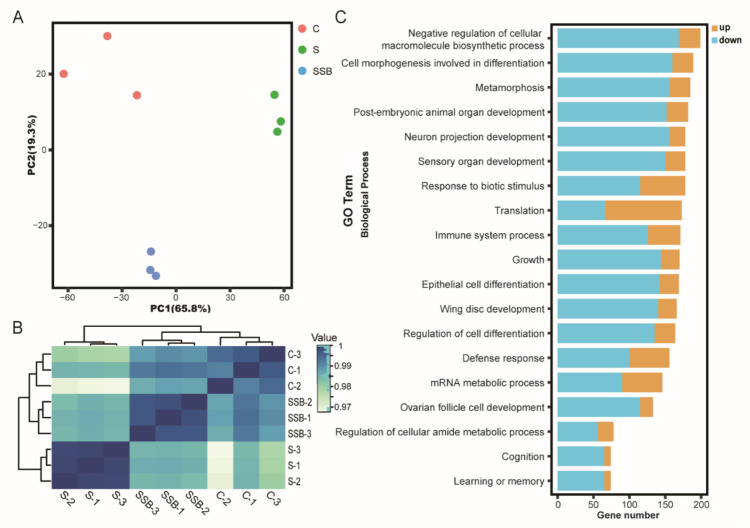
RNA sequencing analysis of conventional *Drosophila* (C), sterile *Drosophila* (S) and sterile *Drosophila* treated with sodium butyrate (SSB). (**A**) Principal component analysis of RNA sequencing data from three groups, *n* = 3. (**B**) Pearson correlation heat map of RNA sequencing data from three groups, white to blue, indicates an increase in correlation. (**C**) Gene ontology enrichment of RNA sequencing data from sterile *Drosophila* compared to sterile *Drosophila* treated with sodium butyrate. The x-axis represents gene numbers upregulated or downregulated, and the y-axis represents different pathways in biological process.

**Figure 2:**
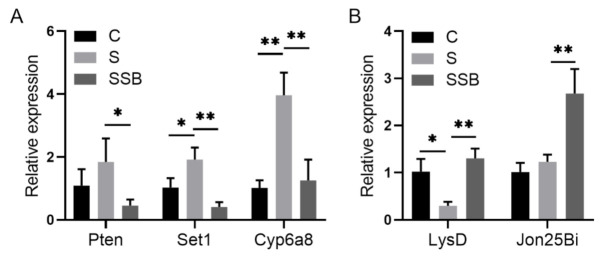
Quantitative RT-PCR validation of genes from sterile *Drosophila* (S) and sterile *Drosophila* treated with sodium butyrate (SSB). (**A**) Relative expression of downregulated transcripts (SSB compared to S) in the transcriptome data. (**B**) Relative expression of upregulated transcripts (SSB compared to S) in the transcriptome data. Significant differences are determined by the unpaired Student’s *t*-test. * *p* < 0.05, ** *p* < 0.01.

Principal component analysis of RNA sequencing data showed that three biological replicates from different groups were well clustered, and samples from conventional *Drosophila* and sterile *Drosophila* were clearly grouped (Figure 1A), suggesting that the sterile *Drosophila* model was successfully established. The Pearson correlation analysis showed gene expression patterns from conventional *Drosophila* and sterile *Drosophila* were clearly different (Figure 1B). As an important microbiota-derived metabolite, sodium butyrate treatment in the sterile *Drosophila* model caused a linear compensation effect between conventional *Drosophila* and sterile *Drosophila* (Figure 1B). A total of 4737 genes were found to be differentially expressed between sterile *Drosophila* and sterile *Drosophila* treated with sodium butyrate. We further analyzed functional pathways with differentially expressed genes; many key pathways in *Drosophila* were affected, such as biosynthetic process, metabolic process, immune response, and development process (Figure 1C). We performed quantitative RT-PCR validation of differentially expressed genes (Figure 2), and the results were generally consistent with RNA sequencing data. Specifically, genes involved in metabolic and immune pathways (both downregulated and upregulated) were differentially expressed between conventional *Drosophila* and sterile *Drosophila* (Figure 2).

### 2.2. Drosophila Microbiome and Metabolites Regulate Host Gene Expression under Conventional Condition

Based on our findings in sterile *Drosophila*, microbiome and microbiota-derived sodium butyrate showed an obvious influence on *Drosophila* gene expression pattern when intestinal microbes were completely removed. We wondered if the effect could be observed when *Drosophila* were grown under conventional conditions, and we were encouraged to explore potential mechanisms underlying the host-microbiome interactions at the molecular level. We treated conventional *Drosophila* with or without sodium butyrate, and we first collected *Drosophila* midgut components for bacterial analysis. 

**Figure 3:**
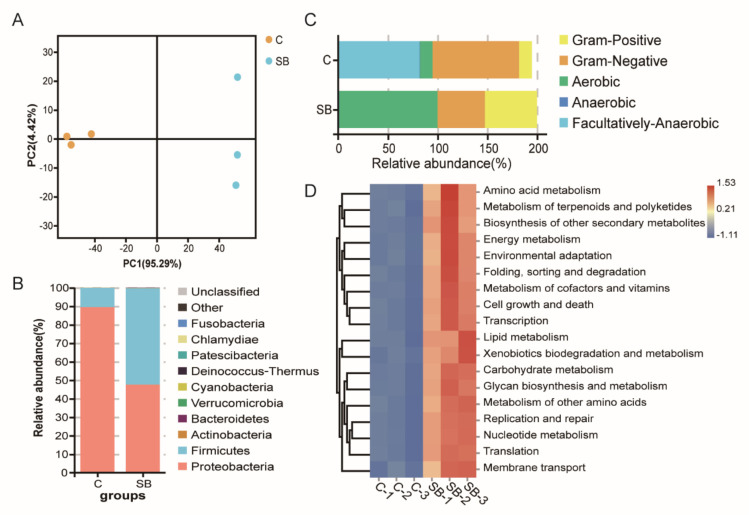
Gut microbiome analysis of 16S rDNA sequencing data from conventional *Drosophila* (C) and sodium butyrate-treated *Drosophila* (SB). (**A**) Principal component analysis of samples from two groups, *n* = 3. (**B**) Microbiome analysis shown at phylum level. (**C**) Organism level microbiome phenotypes predicted with Bugbase. (**D**) Functional prediction heat map with PICRUSt.

For gut microbiome analysis, 16S rDNA sequencing was used to analyze the bacterial structure upon sodium butyrate treatment compared to conventional *Drosophila*. Principal component analysis showed three biological replicates from two groups could be well clustered (Figure 3A), indicating a visible effect of sodium butyrate on *Drosophila* microbiome components. At the phylum level, the dominant effect of sodium butyrate was shown to promote Firmicutes and suppress Proteobacteria (Figure 3B). The bacterial structure was also analyzed at the class, order, family, genus, and species level, respectively (Supplementary Figure S1). Functional prediction of bacterial structure showed that the relative abundance of Gram-positive bacteria was increased and Gram-negative bacteria was decreased by sodium butyrate treatment (Figure 3C), which was consistent with bacterial structure at the phylum level (Figure 3B). In addition, sodium butyrate treatment increased the abundance of aerobic bacteria and decreased the abundance of anaerobic bacteria. Microbiome phenotypes predictions results showed that those changed bacteria species might be involved in many phenotypes with metabolism as the dominant function (Figure 3D).

**Figure 4:**
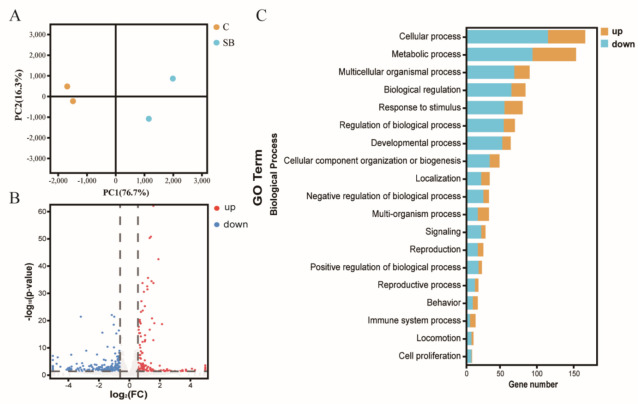
RNA sequencing analysis of conventional *Drosophila* (C) and sodium butyrate-treated *Drosophila* (SB). (**A**) Principal component analysis of samples from two groups, *n* = 2. (**B**) Volcano plot showing transcriptional regulation of genes. (**C**) Gene ontology enrichment of RNA sequencing data from conventional *Drosophila* compared to sodium butyrate-treated *Drosophila*. The x-axis represents gene numbers upregulated or downregulated, and the y-axis represents different pathways in the biological process.

**Figure 5:**
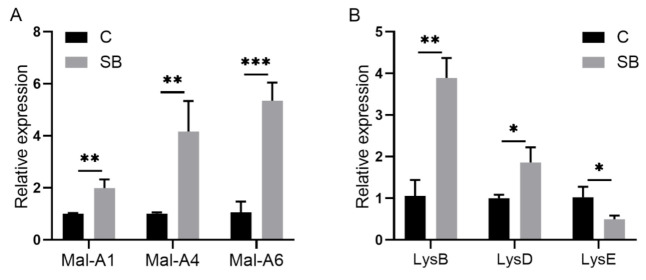
Quantitative RT-PCR validation of genes from conventional *Drosophila* (C) and sodium butyrate-treated *Drosophila* (SB). (**A**) Relative expression of transcripts encoding metabolic enzymes in qRT-PCR experiments. (**B**) Relative expression of transcripts encoding lysozyme family proteins in qRT-PCR experiments. Significant differences are determined by the unpaired Student’s *t*-test. * *p* < 0.05, ** *p* < 0.01, *** *p* < 0.001.

After we confirmed the effect of sodium butyrate on *Drosophila* intestinal microbes, we further performed RNA sequencing of *Drosophila* treated with or without sodium butyrate. Principal component analysis of RNA sequencing data showed that gene expression patterns from different groups could be well clustered (Figure 4A). Volcano plot analysis showed sodium butyrate treatment under conventional conditions could also regulate *Drosophila* gene expression in both up- and downregulation patterns (Figure 4B). The most significant upregulated genes coding for these transcripts were *Met75Ca*, *CG42866*, and the most significant downregulated genes were *CG30025*, *CG33502*, *Cpr49Ah*, and *CG17298*. Gene ontology analysis showed that most differentially expressed genes regulated by sodium butyrate were enriched in key biological processes (Figure 4C). Overall, sodium butyrate treatment showed an inhibitive effect on host gene expression under both conventional conditions (Figure 4C) and sterile conditions (Figure 1C). We also performed quantitative RT-PCR validation of differentially expressed genes, and genes with different functions were selected for validation experiments (Figure 5 and Supplementary Figure S2). The results were similar with RNA sequencing data, suggesting that sodium butyrate treatment for conventional *Drosophila* could affect gene expression, especially genes encoding metabolic enzymes (Figure 5A) and lysozyme family proteins (Figure 5B). 

**Figure 6:**
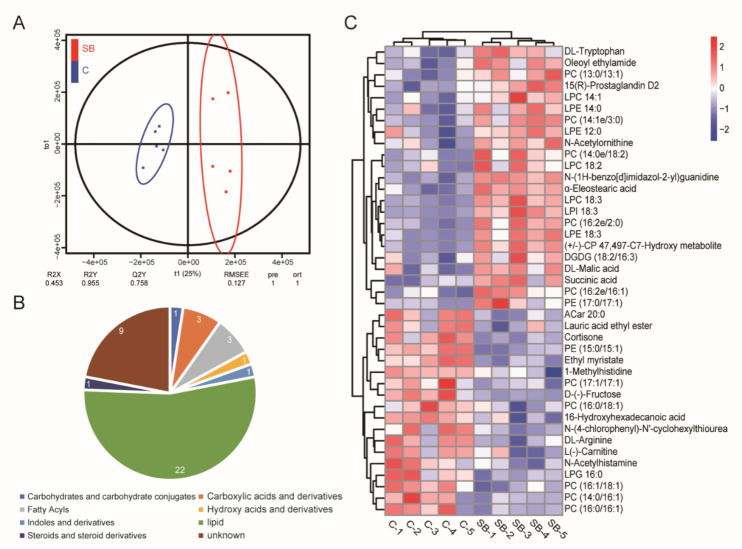
Non-target analysis of metabolites from conventional *Drosophila* (C) and sodium butyrate-treated *Drosophila* (SB). (**A**) Orthogonal partial least squares discriminant analysis of two groups samples, *n* = 5. (**B**) Pie chart showing the proportion of different metabolites components. (**C**) Heat map analysis of metabolites from two groups in both positive and negative electrospray ionization modes. Blue to red indicates an increase in metabolite abundance.

Our gut microbiome data and transcriptome data suggested the dominant role of sodium butyrate in metabolism. Next, we performed non-target analysis using UPLC-MS/MS and obtained the metabolome data of *Drosophila* treated with or without sodium butyrate. Orthogonal partial least squares discriminant analysis showed five biological replicates could be clustered and samples from two groups could be separated (Figure 6A). Pie chart analysis of the proportion of different metabolites components showed lipids were the main components of different metabolites between conventional *Drosophila* and sodium butyrate-treated *Drosophila* (Figure 6B). Heat map analysis of metabolites with different abundance also indicated the clustering of biological replicates between two groups. The results showed that sodium butyrate treatment indeed could influence the abundance of metabolites, with lipids as the dominant change (Figure 6C).

**Figure 7:**
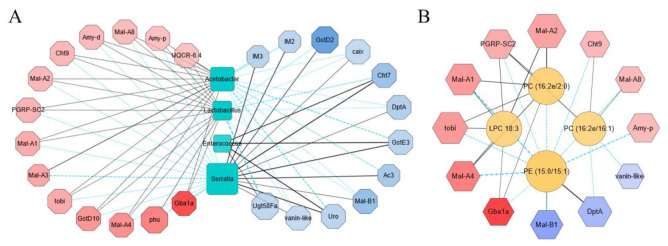
Integrative analysis of the transcriptome, the microbiome, and the metabolome data from conventional *Drosophila* (C) and sodium butyrate-treated *Drosophila* (SB). (**A**) Correlation analysis between the transcriptome and the microbiome data in upregulated and downregulated manner, respectively. (**B**) Correlation analysis between the transcriptome and the metabolome data in an upregulated and downregulated manner, respectively. Upregulated and downregulated transcripts were selected to analyze the correlation with the microbiome and the metabolome data. The correlation analysis was performed using igraph (version 1.1.1) in R package and networks with significant correlations were drawn using Cytoscape software. Red indicates the transcripts with log_2_(fold change) < 0, and blue indicates the transcripts with log_2_(fold change) > 0, an increase of color indicated the extend of fold change. The solid line and dashed line indicated positive correlation and negative correlation, respectively.

To understand the relationship among the transcriptome, the microbiome, and the metabolome with the impact of sodium butyrate, we first carried out a correlation analysis between transcriptome and microbiome with metabolic immunity-related transcripts and the most abundant bacteria in the microbiome analysis results selected in the correlation analysis (Figure 7A). At genus level, the most enriched bacteria affecting the transcriptome pattern were *Acetobacter* and *Lactobacillus*. They were positively correlated with the upregulated genes and negatively correlated with the downregulated genes, while *Serratia* and *Enterococcus* were shown to have the contrary role. Among the upregulated genes, *Gba1a* was involved in lipid metabolism and glycan biosynthesis, while *Mal-A1*, *Mal-A2*, *Mal-A3*, *Mal-A4,* and *Mal-A8* were involved in carbohydrate metabolism and were positively correlated with *Acetobacter* and *Lactobacillus* (Figure 7A). We also performed correlation analysis between transcriptome and metabolome (Figure 7B). The majority of changed metabolites upon sodium butyrate treatment were lipids, such as Phosphatidylcholine (PC), Phosphatidylethanolamine (PE), and lysophosphatidylcholine (LPC). The representative differential metabolites LPC 18:3, PC (16:2e/2:0), PC (16:2e/16:1), and PE (15:0/15:1) were screened out by Cytoscap, and it was obvious that PE (15:0/15:1) was negatively correlated with metabolism-related genes *Mal-A1*, *Mal-A2*, *Mal-A4,* and *Mal-A8*, while the antimicrobial peptide gene *DptA* was positively correlated. Meanwhile, PC (16:2E 2:0), PC (16:2E/16:1), and LPC 18:3 were positively correlated with *PGRP-SC2*, a gene that could promote intestinal immune homeostasis (Figure 7B). 

## 3. Discussion

Host microbiome has been increasingly recognized to play an important role in shaping the health of animals, and the conception that intestinal bacteria communities can affect host physiology has been largely acknowledged [10,11]. Recent advances indicate that the microbiota is involved in the energy balance and metabolic homeostasis of host animals. In mammals, the connection between gut microbiota and energy metabolism is well established to be an area of intense research [12]. However, our current understanding of the impact of gut microbiota on host is generally descriptive, due to technical difficulties associated with integrated analysis of both the microbes and the host [8]. Most research on the impact of the microbiome on animals is designed to identify the effects of single microbial taxa and single metabolite of microbial origin, while the networks of host-microbiome interactions at different layers in nature are much more complicated than we thought [13]. A comprehensive understanding of host-microbiome interactions that integrates multi-omics data information is currently expected in the field.

Recently, there has been growing interest in using *Drosophila melanogaster* to elucidate the mechanisms underlying the complex relationships between the host and its microbiota [14,15], and increasing evidence using *Drosophila* showed that the microbiome can influence many aspects of the host, including the metabolism, the immune system and the behavior [16,17]. In principle, the relative simplicity of the *Drosophila* microbiota makes it a useful model to study host-microbiota interactions. In this context, the fruit fly *Drosophila* is considered a key model for understanding microbiota’s influence on animal health. In the present study, we used *Drosophila* as the model to investigate the impact of microbiome and microbiota-derived metabolite sodium butyrate on host transcriptome and metabolome. Using multi-omics analysis, we found that microbiome and sodium butyrate could impact host gene expression patterns in both sterile *Drosophila* model (Figure 1) and conventional *Drosophila* model (Figure 4). At the molecular level, sodium butyrate is likely to affect host lipid metabolism through gene expression regulation. Our results demonstrated that the microbiome and sodium butyrate of *Drosophila* can significantly alter gene expression, bacterial structure, and metabolites composition of the host. 

Among the short-chain fatty acids from microbiota, sodium butyrate received specific attention because it has been proven to directly activate specific G protein-coupled receptors on enteroendocrine cells and stimulate the synthesis of intestinal endocrine peptides to regulate lipid and carbohydrate metabolism [18,19]. In addition, sodium butyrate was also confirmed as an effective histone deacetylases inhibitor to subsequently regulate the expression of a variety of homeostasis-related genes [20,21]. More importantly, sodium butyrate has been used in aging-related disease therapy since it can improve the memory function in animal models [22]. In our study, we supplemented the *Drosophila* food with sodium butyrate under sterile conditions and conventional conditions. The gene expression patterns of two conditions collectively suggest the effect of sodium butyrate on the host transcriptome with the effect more pronounced in the sterile condition where the background level of sodium butyrate was depleted. Under conventional conditions where *Drosophila* has normal microbiota, sodium butyrate treatment still leads to hundreds of differentially expressed genes and dozens of differential metabolites. Specifically, the most significant upregulated genes are *Met75Ca*, *CG42866*, and the most downregulated genes are *CG30025*, *CG33502*, *Cpr49Ah*, *CG17298*. The microbiome analysis showed that the relative abundance of Gram-positive bacteria was increased and that of Gram-negative bacteria was decreased by sodium butyrate treatment (Figure 3). The results are consistent with the literature in which Gram-positive firmicutes are the main sodium butyrate-producing bacteria [23,24]. In our study, *Acetobacter* and *Lactobacillus* showed a dominant role in the intestinal flora of *Drosophila* treated by sodium butyrate, and the metabolites from these bacteria could be used to maintain an acidic environment in the intestinal tract, thereby inhibiting the growth of harmful bacteria. The negative correlation between these two types of bacteria and antimicrobial peptide genes *IM2*, *IM3,* and *DptA* (Figure 7A) could also support the effect of sodium butyrate on *Acetobacter* and *Lactobacillus*. The metabolites composition in our study showed that sodium butyrate supplement can dramatically cause lipid accumulation (Figure 6); this is also consistent with the previous report that sodium butyrate can regulate lipid metabolism [25,26]. Our work can serve as launching points for future work, and additional investigations are warranted to understand the effects of sodium butyrate on host metabolism, including the specific functions of differentially expressed genes in *Drosophila*.

In this study, we took advantage of *Drosophila* as the model to study host-microbiome interactions and carried out experiments including RNA sequencing, 16S rRNA sequencing, and metabolites profiling. Our integrative analysis of the transcriptome, the microbiome and the metabolome data suggested several candidate transcripts that are coregulated by sodium butyrate. The results from this study provide molecular evidence for host-microbiome interactions with multi-omics data. Our work also validated *Drosophila* as a valuable model for microbiome research, especially as it was very simple to establish the sterile *Drosophila* model with low cost and short time. In our study, we used the entire organism of *Drosophila* for our transcriptome and metabolome. We appreciate that the entire organism may be complex. However, we used the entire organism for both experimental *Drosophila* and control *Drosophila*; thus, the influence of the complexity of different cell types has been minimized. Overall, we believe that the *Drosophila* model will offer a valuable alternative to mammalian models for the fundamental discovery of microbiome functions as indicated in the literature [27,28,29]. Although there are differences between the *Drosophila* and the mammalian model—for example, *Drosophila* lacks an adaptive immune system [30]—the overall immune metabolic pathways that maintain intestinal homeostasis, function, and integrity are still highly conserved [31,32]. Moreover, the host-microbiota interactions are most likely conserved across the animal kingdom and are important for all animal health [31]. The long application record as a successful model for discovering fundamental biological mechanisms has proved *Drosophila* as a valuable system for understanding host-microbiome interactions, and we will continue to carry out systematical investigation on *Drosophila*-microbiome interactions in our future study.

## 4. Materials and Methods

### 4.1. Conventional Drosophila and Sterile Drosophila

For conventional *Drosophila*, *Drosophila melanogaster* w^1118^ was reared at 25 °C under 12 h light/12 h dark cycles on yeast-glucose medium (1 L water, 100 g yeast, 100 g glucose, 1.2% agar, 0.1% potassium sorbate) [33,34]. Sodium butyrate (molecular formula: C_4_H_7_NaO_2_, Sigma, 100 mM dissolved in water) -supplemented food was prepared in yeast-glucose basis medium. Grape juice agar plates were made by microwaving the mixture (100 mL water, 10 g yeast, 10 g glucose, and 1 g of agar), the diet was boiled three times, and grape juice was added to increase the visibility of eggs on the agar plate when we collected eggs [29]. The agar was cooled down and poured into clean Petri dishes, and the yeast was spread on the agar surface. The eggs were collected from the plastic cage by rinsing the agar plate with distilled water and gently poured into the cell strainer. To establish the sterile *Drosophila* model, fresh eggs were dechorionated in 2.7% sodium hypochlorite followed by twice with washes with 70% ethanol for 2 min in the biosafety cabinet. After washing the surface of the eggs with sterile water, a sterilized paintbrush was used to transfer the eggs to a pre-sterilized tube containing food. Sodium butyrate was included in the sterilized food for the treatment group. The protocol for animal experiment was reviewed and approved by the Institutional Animal Care and Use Committee of South China Normal University at Guangzhou, China (protocol code SCNU-SLS-2021-015).

### 4.2. Gut Microbiome Analysis

After 3–4 days of eclosion, *Drosophila* adults were anesthetized with CO_2_ gas followed by surface disinfected with 70% ethanol, washed three times with sterile PBS, and dissected to collect midguts. Equal numbers of males and females were used to ensure no gender differences in the bacterial content (40 flies per replicate). The bacterial DNA was purified using TIANamp Bacteria DNA kit (Tiangen Biotech Inc., Beijing, China), following the manufacturer’s protocols. A region encompassing the V3–V4 hypervariable regions of the 16S rRNA gene was amplified using the primers 341F (CCTACGGGNGGCWGCAG) and 806R (GGACTACHVGGGTATCTAAT). The 16S amplicon sequencing was performed on the Illumina Hiseq2500 platform by Guangzhou Genedenovo Biotechnology. 

For microbiome data analysis, raw data containing adapters or low-quality reads were further filtered using FASTP (https://github.com/OpenGene/fastp, accessed on 7 January 2021), and paired-end clean reads were merged as raw tags using FLSAH [35], with a minimum overlap of 10bp and mismatch error rates of 2%. Clean tags were searched against the reference database to perform reference-based chimera checking using the UCHIME algorithm (http://www.drive5.com/usearch/manual/uchime_algo.html, accessed on 8 January 2021). All chimeric tags were removed, and finally, obtained effective tags were used for further analysis. The effective tags were clustered into operational taxonomic units (OTUs) of  ≥  97% similarity using UPARSE [36]. The tag sequence with the highest abundance was selected as the representative sequence within each cluster. Between-groups Venn analysis was performed in R project (version 3.4.1) to identify unique and common OTUs. The representative sequences were classified into organisms by a naive Bayesian model using RDP [37] based on SILVA database (https://www.arb-silva.de/, accessed on 8 January 2021). Chao1, Simpson, and all other alpha diversity indexes were calculated in QIIME. OTU rarefaction curve and rank abundance curves were plotted in QIIME. Multivariate statistical techniques were calculated and plotted in R project. The KEGG pathway analysis of the OTUs was inferred using PICRUSt [38] and Tax4Fun [39]. Microbiome phenotypes were classified using BugBase. 

### 4.3. RNA Sequencing and Data Analysis

After total RNA was extracted, the enriched mRNA by Oligo(dT) beads were fragmented using fragmentation buffer and reverse transcripted into cDNA with random primers using the NEBNext^®^ Ultra™ RNA library prep kit for Illumina following manufacturer’s instructions. For first-strand cDNA synthesis, the fragmented and primed mRNA was reversed into cDNA using ProtoScript IIReverse Transcriptase in a 20 µL reaction with the procedure of 10 min at 25 °C and 15 min at 42 °C followed by 15 min at 70 °C. For second-strand cDNA synthesis, the Second Strand Synthesis Enzyme Mix was added to the First-Strand Synthesis reaction to react at 16 °C for 1 h in an 80 µL reaction. Then, the cDNA fragments were purified with QiaQuick PCR extraction kit (Qiagen, Germany), end-repaired, poly(A) added, and ligated to Illumina sequencing adapters. The ligation products were size selected by agarose gel electrophoresis, PCR-amplified, and sequenced using Illumina HiSeq2500 by Genedenovo Biotechnology Co, Ltd (Guangzhou, China). For RNA sequencing of *Drosophila* samples under sterile conditions, libraries were sequenced on Illumina HiSeq Xten platform. 

For data processing, quality control of sequencing data was assessed with fastqc (www.bioinformatics.babraham.ac.uk/projects/fastqc/, accessed on 12 January 2021). Adaptor sequences were trimmed by cutadapt [40] with at least 30 nt remaining length, and clean reads were mapped to the dm6 reference genome using HISAT2 [41]. FeatureCounts [42] was used to count the number of reads that mapped to a gene. Gene expression level was calculated by R package DESeq2 [43]. Hierarchical clustering and principal component analysis were used to visualize the effect of different groups. Differentially expressed genes between two groups were identified by the false discovery rate corrected *p*-value < 0.05 and log2|fold change| > 1. In order to obtain the biological annotation of differentially expressed genes, gene ontology analysis was performed by clusterProfiler [44]. The *p*-value < 0.05 and q-value < 0.01 were considered a significant enrichment, and GO (Gene Ontology) terms redundancy were removed by REVIGO [45].

### 4.4. Metabolites Extraction, UHPLC-MS/MS and Metabolome Analysis

*Drosophila* samples three days after feathering were ground separately with liquid nitrogen and homogenized with 80% methanol and 0.1% formic acid precooled resuspended in the vortex well, incubated on ice for 5 min at 4 °C, and centrifuged for 20 min. The supernatant portion was diluted with LC-MS grade water to a final concentration of 53% methanol. The samples were transferred to tubes and centrifuged at 5000× *g* at 4 °C for 20 min. Finally, the supernatant was injected into the LC-MS/MS system for analysis. Ultra-high performance liquid chromatography-MS/MS (UHPLC-MS/MS) was performed using the Vanquish UHPLC system (ThermoFisher Scientific, Waltham, MA, USA) and Orbitrap Q ExactiveTM HF-X mass spectrometer (ThermoFisher) in Genedenovo Biotechnology Co., Ltd.

The raw data files generated by UHPLC-MS/MS were processed using the Compound Discoverer 3.1 (ThermoFisher) to perform peak alignment, peak picking, and quantitation for each metabolite. Peak intensities were normalized to the total spectral intensity. The normalized data were used to predict the molecular formula based on additive ions, molecular ion peaks, and fragment ions; then, the peaks were matched with the mzCloud (https://www.mzcloud.org/, accessed on 20 January 2021) to obtain the accurate qualitative and relative quantitative results. Statistical analyses were performed using the statistical software R, Python and CentOS. 

### 4.5. RNA Preparation and RT-qPCR Analysis

According to the manufacturer’s instructions, *Drosophila* adults were homogenized in 1 mL TRIzol (Accurate Biotechnology, Hunan, China) with sterilized steel balls. The RNA samples were dissolved in an appropriate amount of DEPC water. NanoDrop 2000 spectrophotometer (ThermoFisher) was used to quantify the concentration and purity of total RNA in each sample at a wavelength of 260 nm. The HiFiScript gDNA Removal cDNA Synthesis Kit (CWBIO, Jiangsu, China) was used to synthesize cDNA. Three biological replicates were used for quantitative reverse transcription PCR analysis using MagicSYBR Mixture (CWBIO). The relative mRNA level of gene expression was measured with Rp49 as internal control by calculating the values of ΔCt^Gene^/ΔCt^Rp49^ and analyzed by the 2^−ΔΔCt^ method. Primers used in this study are listed in Supplementary Table S1. 

## 5. Conclusions

This study investigated the role of the microbiome and microbiota-derived metabolite sodium butyrate in regulating host transcriptome and metabolome. To validate our hypothesis, we established both the sterile *Drosophila* model and the conventional *Drosophila* model treated with or without sodium butyrate. Our transcriptome data and qRT-PCR validation results showed that microbiome and sodium butyrate can impact host gene expression patterns in both sterile *Drosophila* model and conventional *Drosophila* model. By 16S rRNA sequencing analysis, we showed that sodium butyrate treatment can affect bacterial structure with the dominant effect on Firmicutes and suppress Proteobacteria. Functional prediction of bacterial structure showed that the relative abundance of Gram-positive bacteria was increased and Gram-negative bacteria was decreased by sodium butyrate treatment, respectively. We also identified *Drosophila* metabolites by ultra-high performance liquid chromatography-MS/MS; the results showed sodium butyrate influenced the composition of host metabolites with lipids as the dominant changed components. Our integrative analysis of the transcriptome, the microbiome, and the metabolome data in this study identified candidate transcripts that are coregulated by sodium butyrate. Taken together, our work reveals the impact of microbiome and microbiota-derived sodium butyrate on host transcriptome and metabolome and provides evidence for a better understanding of host-microbiome interactions with multi-omics data.

## Data Availability

The raw sequencing data analyzed in the current study have been deposited in the NCBI Gene Expression Omnibus database, available under accession number GSE169135.

## Supplementary Materials

The following are available online at https://www.mdpi.com/article/10.3390/metabo11050298/s1, Supplementary Table S1. Primer sequences used for RT-qPCR analysis in this study. Supplementary Figure S1. Gut microbiome analysis of bacterial structure at the class, order, family, genus, species level, respectively. 16S rDNA sequencing data from conventional *Drosophila* (C) and sodium butyrate-treated *Drosophila* (SB) were used, n=3. Supplementary Figure S2. Quantitative RT-PCR validation of genes from conventional Drosophila (C) and sodium butyrate-treated Drosophila (SB). (A) Relative expression of up-regulated transcripts in the transcriptome data. (B) Relative expression of down-regulated transcripts in the transcriptome data. Significant differences are determined by the unpaired Student’s *t*-test. * *p* < 0.05, ** *p* < 0.01.
