# Supplementary material for: The Impact of Microbiome and Microbiota-Derived Sodium Butyrate on Drosophila Transcriptome and Metabolome Revealed by Multi-Omics Analysis
Source: Metabolites. 2021 May 6;11(5):298. doi: 10.3390/metabo11050298 (PMC8148185; doi:10.3390/metabo11050298)
Supplement: Supplementary file 1 [file metabolites-11-00298-s001.zip › metabolites-1169692-supplementary.pdf]

The Impact of Microbiome and Microbiota-Derived Sodium Butyrate on *Drosophila* Transcriptome and Metabolome Revealed by Multi-Omics Analysis

Fan Zhou [1,†], Biaodi Liu [2,†], Xin Liu [1], Yan Li [2], LuoLuo Wang [1], Jia Huang [1], Guanzheng Luo [2,*] and Xiaoyun Wang [1,*]

[1] Guangdong Provincial Key Laboratory of Insect Developmental Biology and Applied Technology, Institute of Insect Science and Technology, School of Life Sciences, South China Normal University, Guangzhou 510631, China.
[2] State Key Laboratory of Biocontrol, School of Life Sciences, Sun Yat-sen University, Guangzhou 510275, PR China.
†These authors have contributed equally to this work.
*Correspondence: luogzh5@mail.sysu.edu.cn; wangxy@scnu.edu.cn

Supplementary Table S1. Primer sequences used for RT-qPCR analysis in this study.

Gene	Forward Primer	Reverse Primer
Pten	AACCTGCTCGGTTGGAACAT	ACACTATCAGCACCACACGAA
Set1	AAAACTTCAACCGCCAGTGC	GGCGGTGCATTTCCAAAAGT
Cyp6a8	GAAAGCAAGGCACCGTTTGT	GGCACCGTCCAGATTGAAGA
LysD	CTGCAACGCTCTCCTGACC	TGCGCAATCGTTTTAATCGGG
Jon25Bi	GCGGTGGTTCTATCATCGCC	GGCCAGTTGTGGTTCTGGAT
Mal-A1	AGTGGTGGGAGAGTGGAAACT	GCCGATGTCTTTCAGGTACTG
Mal-A4	TTCAAAGACAGTGACGGAAATGG	TTTCAGAAAAGGAGACAGCCAG
Mal-A6	GCAGCGATACTCGTTTTGGG	TGCCACTTGCCACCAATCTC
Gba1a	CAGGGACTTCGGTTCTCCAC	GACAGGATTGTCGCATTCGC
LysB	TCTTGACCGACGACATCACC	CGAAGAGGAGATGCTGGTGTTA
LysE	TAAGGGCCCATTGCGAGGA	CCAATTGGTCACGAGGAACG
SPH93	GCAATCGGAACTCTACTCTTAGC	TGGAGCGGTAATCAAATGTTGG
Hsp23	ATTCCATTGTTGTTGAGCCTTGC	GGCCAAGTAGGGATTCCTCTG
Hsp68	CGACAACGGCAAACCAAAGAT	TAGTGCCCAGGTAGGCTTCTG
Lip2	CGGCAGCACATCGACTTGA	GGAGTGTCCAATTAGTACCACCT
CG6283	GCTCCGTTCTCTACTACGCC	GTAGGTGCTTCCGCAGTTCT
CG5246	CTGTCTCAATGTTCGGCGAAA	CCTCCAATCACACGGGTCTC
Jon25Bi	GCGGTGGTTCTATCATCGCC	GGCCAGTTGTGGTTCTGGAT
Npc2e	CACTTCCTGGGCAATAACAACA	GACACTTCGTCGGGTAGGG
Intr	GACCGCTCCGCAGTTTAATGT	CTCTGGTAGGGATAGGGCACT
Lectin-37Da	AAACCCGTACAACTTGACCGT	TTCTCGTAGGCGACATACCAA
Gba1a	CAGGGACTTCGGTTCTCCAC	GACAGGATTGTCGCATTCGC
Ctr1B	TGGTGTCCTTCCTGTATGAGG	CTCGTTCTTCCTACGCTGCT
SPH93	GCAATCGGAACTCTACTCTTAGC	TGGAGCGGTAATCAAATGTTGG
Rp49	GCCCAAGGGTATCGACAACA	ACCTCCAGCTCGCGCACGTT

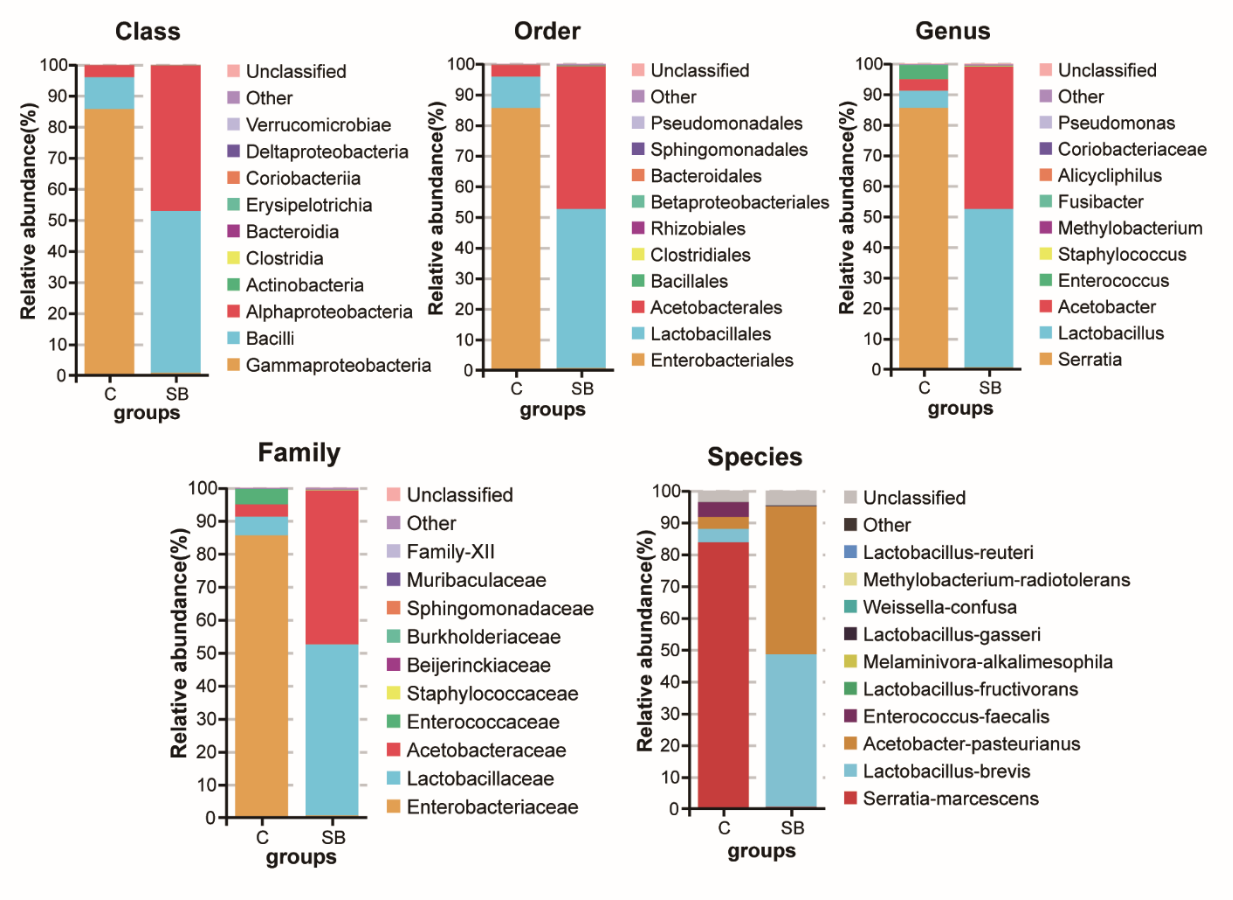

Supplementary Figure S1. Gut microbiome analysis of bacterial structure at the class, order, family, genus, species level, respectively. 16S rDNA sequencing data from conventional *Drosophila* (C) and sodium butyrate-treated *Drosophila* (SB) were used, *n*=3.

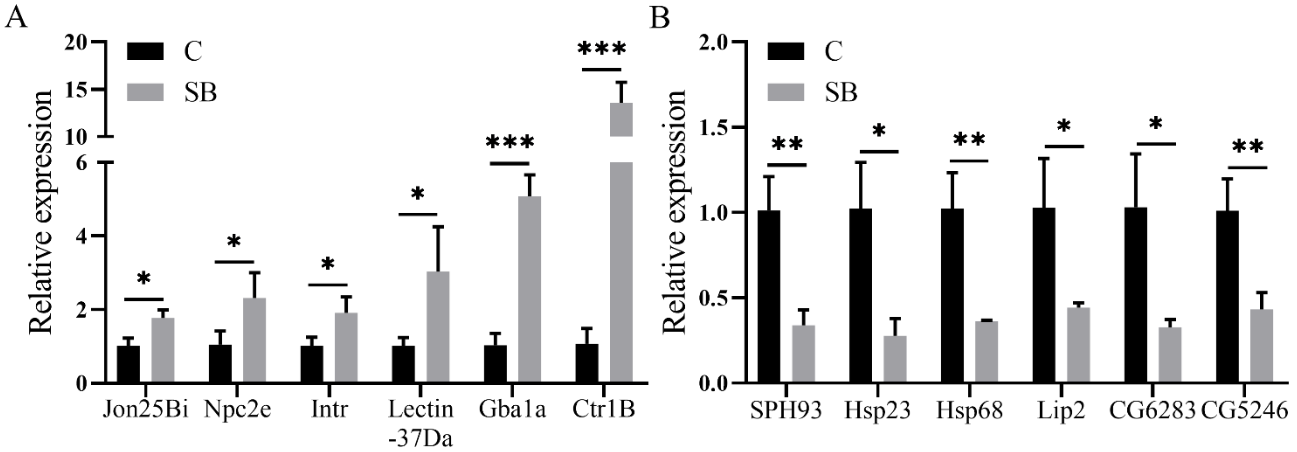

Supplementary Figure S2. Quantitative RT-PCR validation of genes from conventional *Drosophila* (C) and sodium butyrate-treated *Drosophila* (SB). (**A**) Relative expression of up-regulated transcripts in the transcriptome data. (**B**) Relative expression of down-regulated transcripts in the transcriptome data. Significant differences are determined by the unpaired Student's *t*-test. $^{*}P < 0.05$, $^{**}P < 0.01$.
